# Pirfenidone attenuates cardiac hypertrophy against isoproterenol by inhibiting activation of the janus tyrosine kinase-2/signal transducer and activator of transcription 3 (JAK-2/STAT3) signaling pathway

**DOI:** 10.1080/21655979.2022.2073145

**Published:** 2022-05-24

**Authors:** Zhenhuan Chen, Haiwen Zhou, Xiantao Huang, Shaoyun Wang, Xiaochao Ouyang, Yunxia Wang, Qianqiang Cao, Liu Yang, Yu Tao, Hengli Lai

**Affiliations:** Department of Cardiology, Jiangxi Provincial People's Hospital, The First Affiliated Hospital of Nanchang Medical College, Nanchang, Jiangxi, China

**Keywords:** Pirfenidone, cardiac hypertrophy, JAK-2/STAT-3, inflammation

## Abstract

Cardiovascular risk factors have attracted increasing attention in recent years with the acceleration of population aging, amongst which cardiac hypertrophy is the initiating link to heart failure. Pirfenidone is a promising agent for the treatment of idiopathic pulmonary fibrosis and has recently proven to exert inhibitory effects on the inflammatory response. This study proposes to explore the potential pharmacological action of pirfenidone in treating cardiac hypertrophy in a rodent model. Four groups of mice were used in the present study: the control, ISO (5 mg/kg/day) for 7 days, pirfenidone (200 mg/kg/day) for 14 days, and spironolactone (SPI) (200 mg/kg/day) for 14 days groups. Increased heart weight index, left ventricle (LV) weight index, LV wall thickness, declined LV volume, and elevated serum levels of CK-MB, AST, and LDH were observed in ISO-challenged mice, all of which were dramatically reversed by the administration of pirfenidone or SPI. Furthermore, an elevated cross-sectional area of cardiomyocytes in the wheat germ agglutinin (WGA) staining of heart cross-sections, upregulated atrial natriuretic peptide (ANP), brain natriuretic peptide (BNP), β-Myosin Heavy Chain (β-MHC), and excessively released tumor necrosis factor-α (TNF-α) and interleukin 6 (IL-6) in cardiac tissues were observed in the ISO group but greatly alleviated by pirfenidone or SPI. Lastly, the promoted expression levels of p-JAK-2/JAK-2 and p-STAT3/STAT-3 in the cardiac tissues of ISO-challenged mice were significantly repressed by pirfenidone or SPI. Collectively, our data reveals a therapeutic property of pirfenidone on ISO-induced cardiac hypertrophy in mice.

## Graphical abstract

**Figure:**
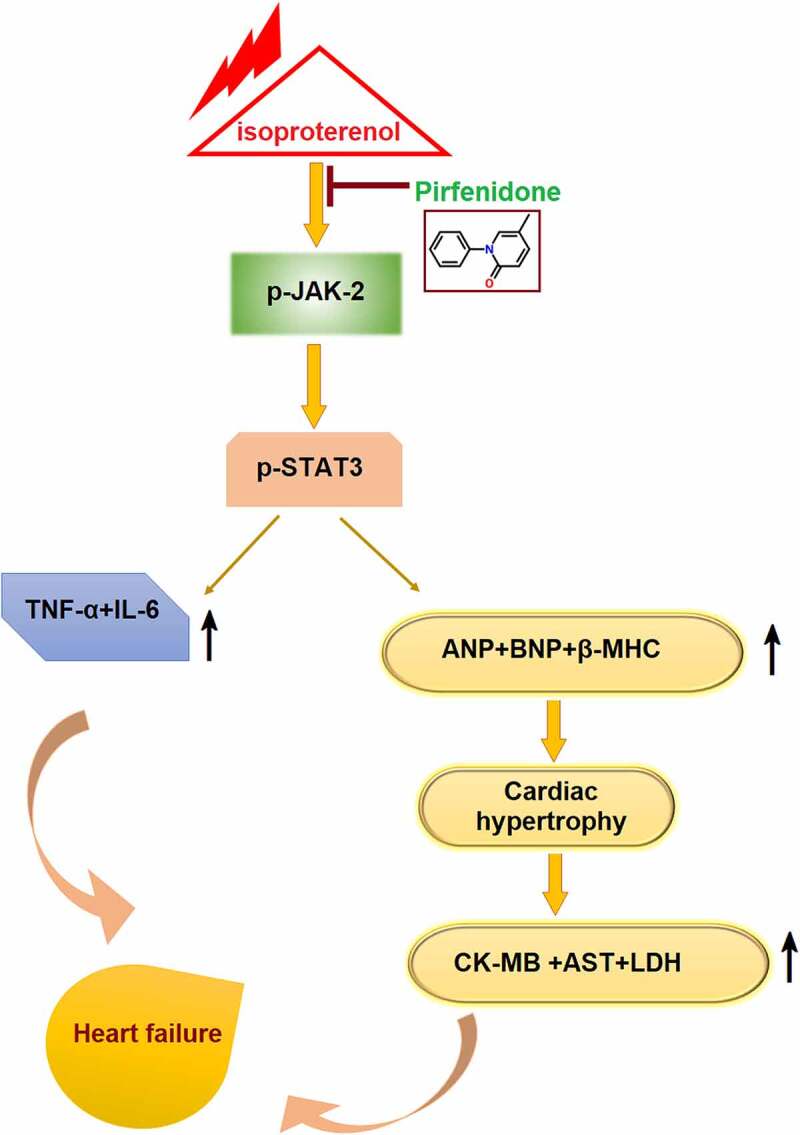

## Highlights

- Pirfenidone ameliorates ISO-induced increases in heart weight;
- Pirfenidone reduced the levels of CK-MB, AST, and LDH;
- Pirfenidone attenuated cardiac hypertrophy in ISO- challenged mice;
- Pirfenidone prevents the activation of the JAK-2/STAT-3 signaling pathway

## Introduction

In recent years, the incidence of cardiovascular risk factors such as hypertension, valvular heart disease, and coronary atherosclerotic heart disease increases annually, resulting in high incidence and mortality of stress-induced heart failure and heart failure after myocardial infarction. Statistically, approximately 4 million patients aged 35–74 years are suffering from heart failure and the morbidity of chronic heart failure is 0.9%, with a 5.4% 30-day mortality in hospitals. In the elderly population, the 5-year survival rate of heart failure is close to that of malignant tumors [1]. Therefore, it is important to elucidate the pathogenesis and endogenous protective factors of heart failure to prevent its progression. It has been confirmed that ventricular remodeling is the basic pathological mechanism of heart failure, the main characteristics of which include pathological hypertrophy of cardiac myocytes, myocardial cell apoptosis, proliferation, and fibrosis of myocardial interstitial fibroblasts [2,3]. Cardiomyocyte hypertrophy is the initiating link to heart failure induced by cardiac remodeling and decreased myocardial contractility [4,5]. In the early stage of heart failure, several factors, such as cardiac valvular disease, hypertension, and myocardial infarction, contribute to cardiac hypertrophy and increased cardiac load. In cardiomyocytes, compensatory adaptive responses are kept to maintain or increase cardiac output, which results in cardiac hypertrophy in the long term. As a consequence, apoptosis of cardiac myocytes and decreased cardiac contractility are induced [6]. Specific molecular and phenotypic changes in cardiac hypertrophy are reported. For example, reactivated atrial natriuretic peptide (ANP), brain natriuretic peptide (BNP), and β Myosin Heavy Chain (β-MHC), upregulated c-Jun, c-fos, and c-myc, and increased protein synthesis, cell volume, and muscle fiber capacity [7]. However, the specific mechanisms underlying these molecular and phenotypic changes remain unclear. Current studies on inflammatory response and cardiovascular disease show that in the occurrence and development of various cardiovascular diseases, excessive release of various inflammatory factors is observed, accompanied by the infiltration of inflammatory cells, which plays an important role in the progression of atherosclerosis, the onset of acute coronary syndrome, deterioration of heart failure, and ventricular remodeling [8,9]. β-adrenergic receptors (β-receptors) are the major receptors regulating cardiac function, while isoproterenol (ISO) is a stimulator of β-receptors and is reported to induce the production of inflammatory factors in myocardial cells [10]. It is, therefore, widely used to establish animal models of cardiac hypertrophy [11].

Pirfenidone is one of the two drugs currently approved in the European Union for the treatment of idiopathic pulmonary fibrosis. It is used to improve lung function, reduce mortality, and prolong progression-free survival in patients with mild to moderate pulmonary fibrosis [12]. Pirfenidone plays an anti-fibrosis role through a variety of mechanisms, including down-regulating the expressions of transforming growth factor β (TGF-β) and tumor necrosis factor α (TNF-α), inhibiting the inflammatory response, and reducing the activation of myofibroblasts [13]. However, the protective effects of pirfenidone in cardiac hypertrophy have not been reported before. This study proposes to explore the potential pharmacological action of pirfenidone in treating cardiac hypertrophy.

## Materials and methods

### Animals and grouping

**Figure 1.**
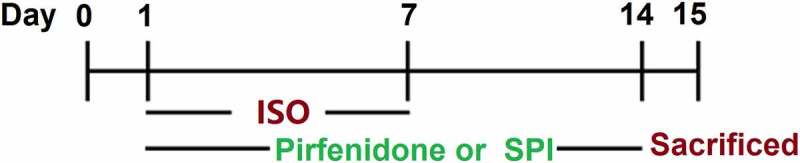
Flow chart of the experimental design.

The animal use protocol in the study has been reviewed and approved by the independent Animal Ethical and Welfare Committee of Jiangxi Provincial People’s Hospital, The First Affiliated Hospital of Nanchang Medical College (No. NMCH-E-2018a021). Forty male C57BL/6 mice were obtained from GemPharmatech (Jiangsu, China) and were divided into 4 groups: the control group: received normal saline; ISO group: the animals were submitted to subcutaneous (sc) injections of ISO (5 mg/kg/day) for 7 days; pirfenidone group: mice were given pirfenidone by gavage for 14 days;

Spironolactone (SPI) group: mice were given SPI by gavage for 14 days. The experimental design is shown in Figure 1 [14].

### Echocardiography

After anesthetization using the 2% isoflurane, the cardiac function of mice was evaluated using the echocardiography with a Motion mode diasonograph (Doppler, New York, USA). In the processing of diastole and contraction of the heart, the left ventricle (LV)’s diastolic anterior wall thickness (LVAWd), LV systolic anterior wall thickness (LVAWs), LV diastolic posterior wall thickness (LVPWd), LV systolic posterior wall thickness (LVPWs), LV end-diastolic volume (LVEDV), and LV end-systolic volume (LVESV) were detected to evaluate the myocardial function of animals.

### Real-time PCR assay

The total RNAs were extracted from cardiac tissues with a TRIzol reagent, followed by being transcribed into cDNA using the HiScript II Q RT SuperMix for qPCR (+gDNA wiper) kit (Vazyme, Jiangsu, China). The PCR reaction conducted in the present study was performed using the 2× SYBR Green PCR Master Mix (Lifeint, Fujian, China). Finally, the relative expression level of target genes was determined using the 2^−ΔΔCt^ method after normalization with the expression of GAPDH [15]. Primers used in this study are listed in Table 1.

**Table 1.** Primer sequences

Target gene	Upstream Sequence (5’-3’)	Downstream Sequence (5’-3’)
ANP	CCTGTGTACAGTGCGGTGTC	AAGCTGTTGCAGCCTAGTCC
BNP	ACAGAAGCTGCTGGAGCTGA	CCGATCCGGTCTATCTTGTG
β-MHC	TATCGATGACCTGGAGCTGA	AGTATTGACCTTGTCTTCCTC
GAPDH	TGACCTCAACTACATGGTCTACA	CTTCCCATTCTCGGCCTTG

### Western blot analysis

The BCA kit (Cwbio, Jiangsu, China) was utilized to quantify the protein isolated from cardiac tissues, which were further separated with the 12% SDS-PAGE. The separated protein was then transferred from the gel to the PVDF membrane, and mixed with 5% skim milk. Then, the membrane was introduced with the primary antibody against p-JAK-2 (1:1000, Santa Cruz Biotechnology, USA), JAK-2 (1:2000, Santa Cruz Biotechnology, USA), p-STAT-3 (1:1000, Santa Cruz Biotechnology, USA), STAT-3 (1:2000, Santa Cruz Biotechnology, USA), and β-actin (1:8000, Santa Cruz Biotechnology, USA). The secondary antibody (1:2000, SolelyBio, Beijing, China) was subsequently added to be incubated for 90 min. Finally, the ECL reagent was added to expose the bands, which were further quantified with the Image J software [16].

### Enzyme-linked immunosorbent assay (ELISA)

The release of TNF-α and IL-6 in cardiac tissues was checked using the commercial ELISA kits (Mlbio, Beijing, China). The homogenate of cardiac tissues was obtained, followed by centrifugation and collecting of the supernatant. ELISA plates were coated with 100 µL coating buffer at 4°C overnight, followed by incubation with 200 µL blocking buffer. Samples were then implanted in a 96-well plate together with the standards for 2 hours. The detection antibody was then added and incubated for 2 hours. Following incubation at 37°C for 60 min, the medium was removed and diluted streptavidin-HRP reagent was introduced into the wells. After incubation at 37°C for 60 min, TMB solution was added to the plates and incubated for 15 min, followed by adding the stop solution. Lastly, the microplate reader (LIUYI BIOTECHNOLOGY CO.,LTD, Beijing, China) was used to obtain the OD value at 450 nm.

### Measurement of biomarkers

The serum was obtained from isolated blood after centrifugation and the concentrations of CK-MB, AST, and LDH in the serum were measured using the automatic biochemical analyzer (7600, Hitachi, Tokyo, Japan).

### Wheat germ agglutinin (WGA) staining

Cardiac tissues were isolated and fixed in 4% paraformaldehyde, followed by being cut into slices and embedded with paraffin. After dewaxing, slides were batched for 20 min at 80°C, followed by being washed with D-Hanks solution twice. Then sections were stained with WGA working solution and incubated at room temperature for 10 min. After being sealed with glycerin, slides were observed and photographed under a fluorescence microscope.

## Statistical analysis

Mean ± standard deviation (S.D.) was presented for each assay and data were analyzed using the analysis of variance (ANOVA) method with the software of GraphPad prism software 6.0. P < 0.05 was considered to be a statistically significant difference.

## Results

In our study, our results demonstrate that pirfenidone treatment dramatically reversed increased heart weight index, left ventricle (LV) weight index, LV wall thickness, declined LV volume, and elevated serum levels of CK-MB, AST, and LDH in ISO- challenged mice. Furthermore, pirfenidone attenuated cardiac hypertrophy and inflammatory response in the cardiac tissues of ISO- challenged mice. Importantly, pirfenidone prevented the activation of the JAK-2/STAT-3 signaling pathway.

### Pirfenidone ameliorated ISO-induced increases in heart weight

**Figure 2.**
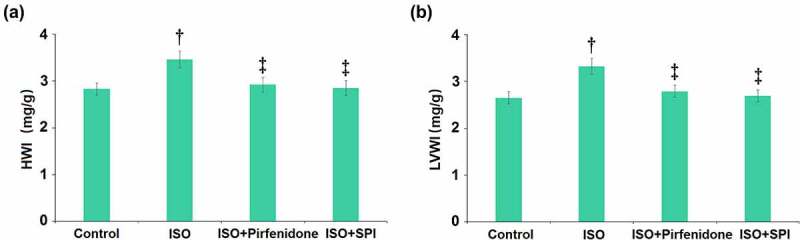
Pirfenidone ameliorates ISO-induced increases in heart weight. (a). Heart weight index (HWI); (b). Left ventricle weight index (LVWI) (†, p < 0.01 vs. Vehicle group; ‡, p < 0.01 vs. ISO group). n = 5 in each group.

We first checked the heart and body weight of each animal. Spironolactone (SPI) was used as a positive control. The heart weight index (Figure 2a) was found significantly increased from 2.83 mg/g to 3.46 mg/g in the ISO group, which was then decreased to 2.92 mg/g by pirfenidone and 2.85 mg/g by SPI. Furthermore, the LV weight index (LVWI) in the ISO group (Figure 2b) was greatly elevated from 2.65 mg/g to 3.32 mg/g, then repressed to 2.79 mg/g and 2.69 mg/g by pirfenidone and SPI, respectively. These data suggest that the increased heart weight in mice induced by ISO was inhibited by pirfenidone.

### Pirfenidone decreased LV wall thickness and increased LV volume in mice

**Figure 3.**
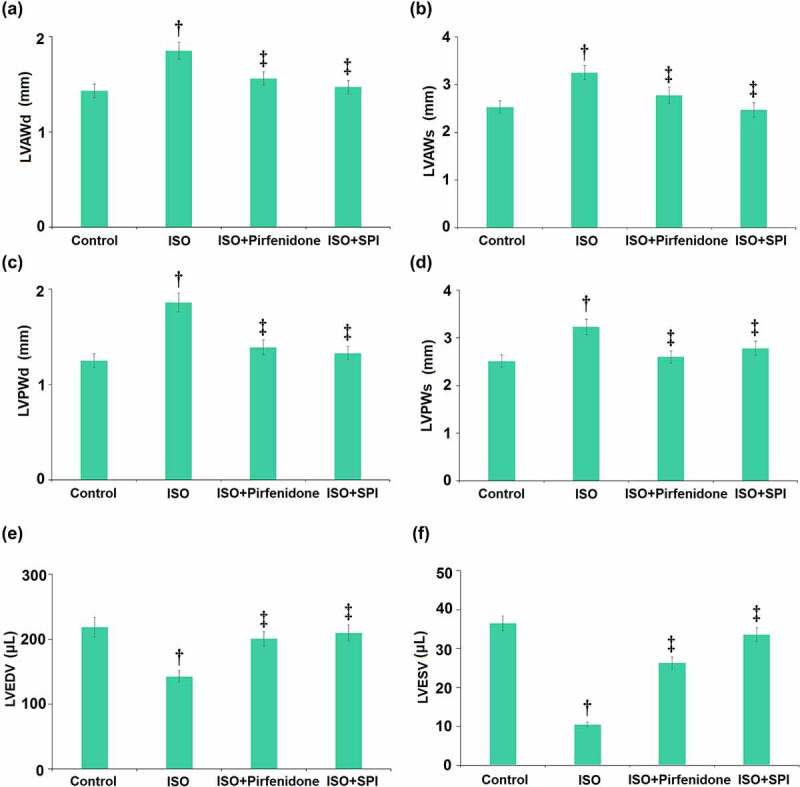
Pirfenidone decreases left ventricle (LV) wall thickness and increases LV volume in mice. Left ventricle (LV)’s diastolic anterior wall thickness (LVAWd), LV systolic anterior wall thickness (LVAWs), LV diastolic posterior wall thickness (LVPWd), LV systolic posterior wall thickness (LVPWs), LV end diastolic volume (LVEDV), and LV end systolic volume (LVESV) (†, p < 0.01 vs. Vehicle group; ‡, p < 0.01 vs. ISO group). n = 5 in each group.

The morphology of cardiac tissues was further checked by echocardiography (Figure 3). LVAWd in the control, ISO, pirfenidone, and SPI groups was 1.43, 1.85, 1.56, and 1.47 mm, respectively. LVAWs was elevated from 2.53 mm to 3.25 mm by ISO, then declined to 2.78 mm and 2.47 mm in the pirfenidone and SPI groups, respectively. Furthermore, LVPWd was increased from 1.25 mm to 1.86 mm in the ISO group, then dramatically reduced to 1.39 mm and 1.33 mm by pirfenidone and SPI, respectively. LVPWs in the control, ISO, pirfenidone, and SPI groups was 2.51, 3.23, 2.6, and 2.78 mm, respectively. LVEDV in the control, ISO, pirfenidone, and SPI groups was 218.32, 142.6, 200.6, and 209.8 μL, respectively. Lastly, LVESV was declined from 36.5 μL to 10.5 μL in the ISO group, then dramatically reversed to 26.3 μL and 33.6 μL by pirfenidone and SPI, respectively. These data suggest that the altered LV wall thickness and LV volume in ISO-treated mice were reversed by pirfenidone.

### Pirfenidone modulated hemodynamic parameters in ISO-challenged mice

**Figure 4.**
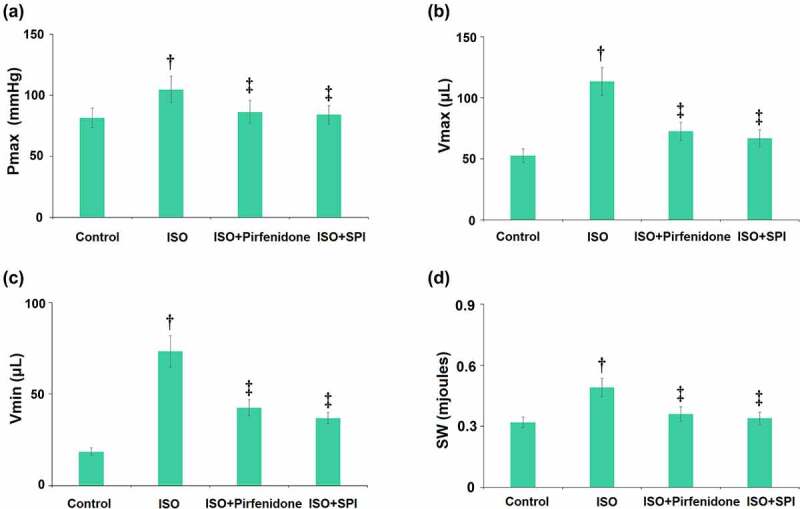
Pirfenidone modulates hemodynamic parameters in ISO-challenged mice. (a). Quantification of left ventricular pressure (Pmax) (b). Quantification of volume (Vmax); (c). Quantification of volume (Vmin); (d). Stroke work (SW) (†, p < 0.01 vs. Vehicle group; ‡, p < 0.01 vs. ISO group). n = 5 in each group.

Hemodynamic parameters were then analyzed. Results demonstrate that ISO significantly elevated the left ventricular pressure (Pmax), volume (Vmax and Vmin), and stroke work (SW). However, the administration of pirfenidone significantly reduced the increases in the Pmax, Vmax, Vmin, and SW (Figure 4).

### Pirfenidone reduced the levels of myocardial injury markers in ISO-challenged mice

**Figure 5.**
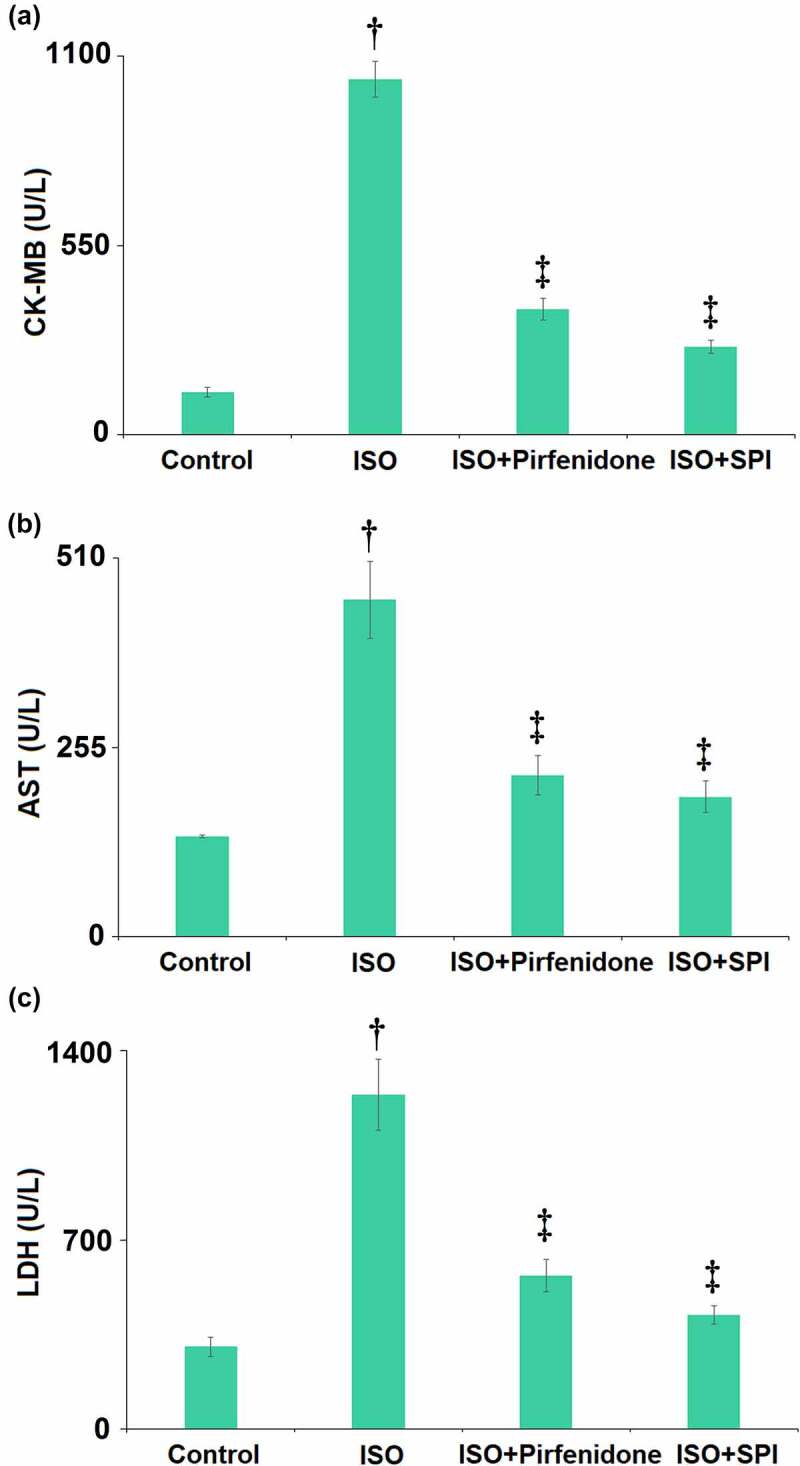
Pirfenidone reduced the levels of myocardial injury markers in ISO- challenged mice. (a) Serum levels of CK-MB; (b) Serum levels of AST; (c) Serum levels of LDH (†, p < 0.01 vs. Vehicle group; ‡, p < 0.01 vs. ISO group). n = 5 in each group.

The levels of myocardial injury markers in the serum were detected using the automatic biochemical analyzer. The serum level of CK-MB (Figure 5a) was dramatically promoted from 123.5 U/L to 1032.6 U/L by ISO, then declined to 365.7 U/L and 256.8 U/L by pirfenidone and SPI, respectively. The concentrations of ALT (Figure 5b) in the serum in the control, ISO, pirfenidone, and SPI groups were 135.2, 453.6, 217.5, and 188.6 U/L, respectively. Furthermore, the release of LDH (Figure 5c) was elevated from 305.8 U/L to 1236.6 U/L, then reduced to 568.9 and 423.5 U/L by pirfenidone and SPI, respectively. These results reveal that the levels of myocardial injury markers in ISO-challenged mice were repressed by pirfenidone.

### Pirfenidone attenuated cardiac hypertrophy in ISO-challenged mice

**Figure 6.**
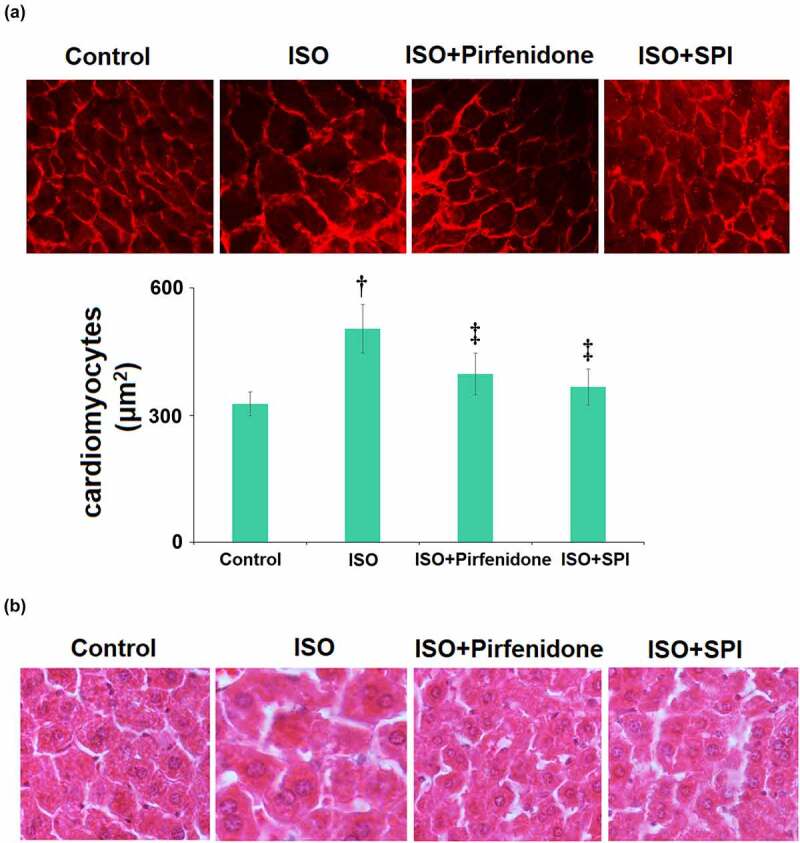
Pirfenidone attenuated cardiac hypertrophy in ISO-challenged mice. (a). Representative images of wheat germ agglutinin (WGA) staining of heart cross-sections and the cross-sectional area of cardiomyocytes; (b). H&E staining to investigate cardiac histopathological changes (†, p < 0.01 vs. Vehicle group; ‡, p < 0.01 vs. ISO group). n = 5 in each group.

The pathological changes in cardiac tissues were checked by WGA staining assay. The representative images of WGA staining of heart cross-sections are visualized in Figure 6a. The cross-sectional area of cardiomyocytes in the ISO group was dramatically promoted from 326.6 μm^2^ to 503.8 μm^2^, then reduced to 397.5 and 366.3 μm^2^ by pirfenidone and SPI, respectively, indicating a protective effect of pirfenidone against ISO- induced cardiac hypertrophy in mice.

Next, we measured the myocardial injury and cardiac remodeling using H&E staining. Results in Figure 6b indicate that the LV tissues in the ISO group exhibited clearly disrupted structure, and the cardiomyocyte surface area was significantly larger than that in the control group. However, these characteristic changes induced by ISO were significantly improved by the administration of pirfenidone and SPI.

### Pirfenidone decreased the gene expression levels of hypertrophic markers ANP, BNP, and β-MHC in ISO-challenged mice

**Figure 7.**
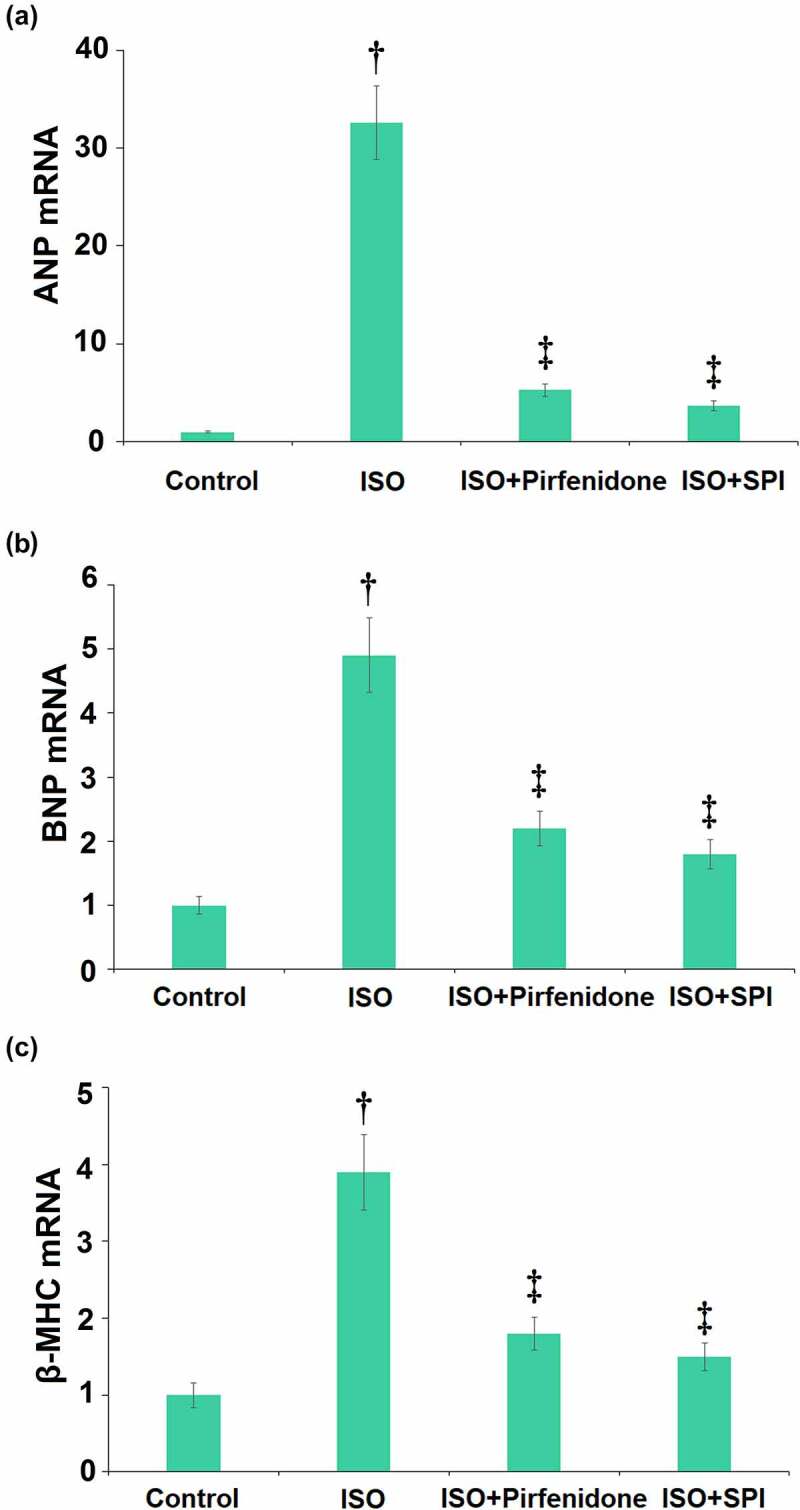
Pirfenidone decreases the mRNA levels of hypertrophic markers ANP, BNP, and β-MHC in ISO-challenged mice. (a). the mRNA levels of ANP; (b). the mRNA levels of BNP; (c). the mRNA levels of β-MHC (†, p < 0.01 vs. Vehicle group; ‡, p < 0.01 vs. ISO group). n = 5 in each group.

The pathological biomarkers of cardiac hypertrophy in cardiac tissues were measured using the real-time PCR assay. As illustrated in Figure 7, ANP, BNP, and β-MHC in the cardiac tissues were greatly upregulated in ISO-treated mice, then dramatically downregulated by pirfenidone and SPI.

### Pirfenidone suppressed inflammatory response in cardiac tissues of ISO-challenged mice

**Figure 8.**
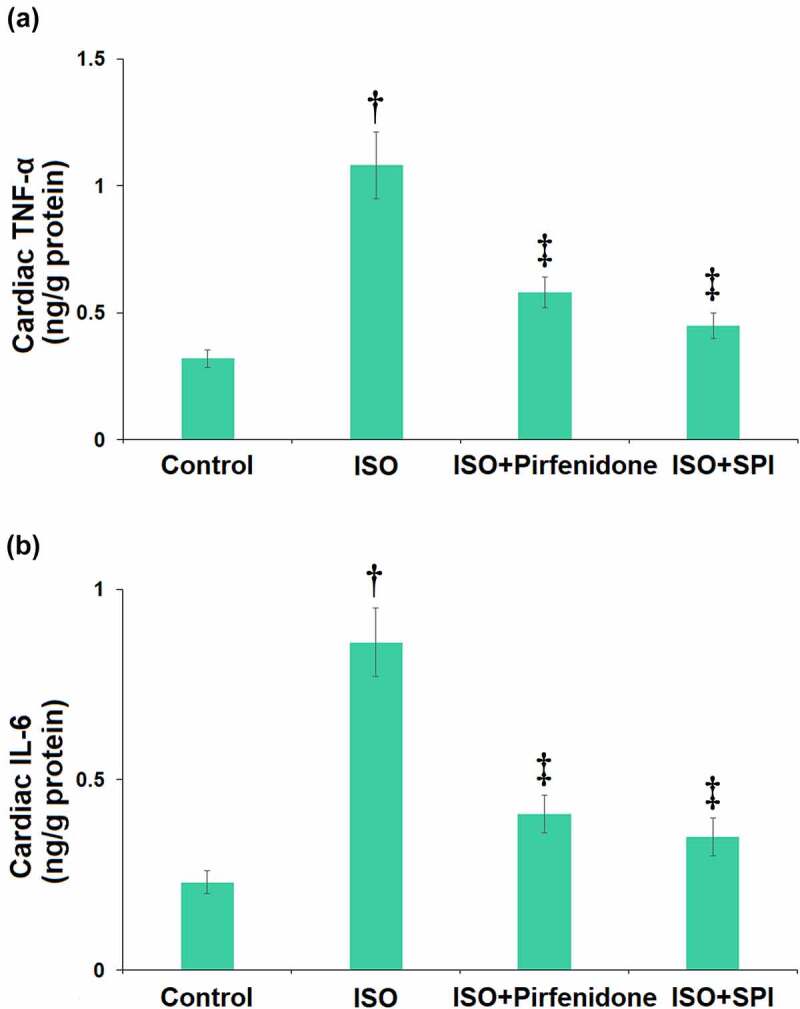
Pirfenidone suppresses inflammatory response in cardiac tissues of ISO-challenged mice. (a) Cardiac TNF-α as measured by ELISA; (b) Cardiac IL-6 as measured by ELISA (†, p < 0.01 vs. Vehicle group; ‡, p < 0.01 vs. ISO group). n = 5 in each group.

Inflammation is an important risk for the development of cardiac hypertrophy. We found that the concentrations of cardiac TNF-α in the control, ISO, pirfenidone, and SPI groups were 0.32, 1.08, 0.58, and 0.45 ng/g protein, respectively. Furthermore, the level of cardiac IL-6 was elevated from 0.23 ng/g protein to 0.86 ng/g protein by ISO, then reduced to 0.41 and 0.35 ng/g protein by pirfenidone and SPI, respectively (Figure 8). These data suggest that the inflammatory response in the cardiac tissues of ISO-challenged mice was significantly repressed by pirfenidone.

### Pirfenidone prevented the activation of the JAK-2/STAT-3 signaling pathway in cardiac tissues of ISO-challenged mice

**Figure 9.**
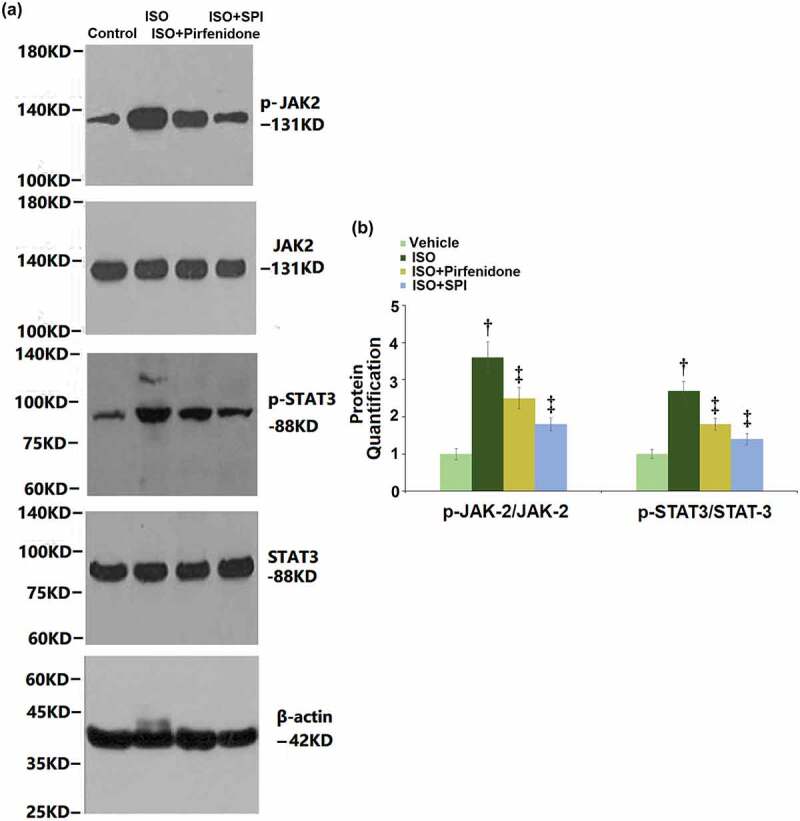
Pirfenidone prevents the activation of the JAK-2/STAT-3 signaling pathway in cardiac tissues of ISO-challenged mice. (a). Western blot analysis of p-JAK-2, JAK-2, p-STAT-3, STAT-3, and β-actin; (b). Quantification of p-JAK-2/JAK-2 and p-STAT3/STAT-3 (†, p < 0.01 vs. Vehicle group; ‡, p < 0.01 vs. ISO group). n = 5 in each group.

The JAK/STAT signaling pathway is found activated in the progression of cardiac hypertrophy [17]. The expression levels of key proteins in the JAK/STAT signaling pathway were determined by western blotting (Figure 9). We found that the levels of p-JAK-2/JAK-2 and p-STAT3/STAT-3 were dramatically promoted in the ISO group, but greatly inhibited by pirfenidone and SPI, indicating an inhibitory effect of pirfenidone on the JAK-2/STAT-3 signaling pathway in cardiac tissues of ISO- challenged mice.

## Discussion

Cardiac hypertrophy has been identified as a predictor of cardiovascular morbidity and mortality and is associated with cardiomyocyte hypertrophy at the cellular level and ventricular remodeling of the heart. Cardiac hypertrophy results from the physiological compensatory response to pathological hypertrophy and fibrosis under the action of various pathogenic factors [18,19]. The pathological manifestations of cardiac hypertrophy include hypertrophy of myocardial cells, hyperplasia of myocardial interstitium, myocardial weight increase, hypertrophy of ventricular walls, and decreased ventricular lumen volume, all of which are important processes of rational reconstruction of heart disease [20]. In the early stage, cardiac hypertrophy is regarded as a compensatory reaction. However, as the disease progresses, serious cardiovascular accidents, such as acute myocardial infarction, heart failure, arrhythmias, and even sudden death, are triggered in the decompensated stage [21]. Studies have shown that cardiac hypertrophy is closely associated with sympathetic excitation and the α or β adrenergic receptor agonists are reported to induce cardiac hypertrophy [22,23]. Repeated or continuous application of ISO, a β adrenoceptor agonist, contribute to the development of cardiac hypertrophy, which is one of the most common methods for establishing experimental models of cardiac hypertrophy [24]. The present study utilized ISO to establish cardiac hypertrophy models in mice, which was verified by increased heart weight and LV wall thickness, as well as decreased LV volume. These pathological changes in cardiac morphology are consistent with the description reported previously [25]. Furthermore, increased levels of myocardial injury markers, the elevated cross-sectional area of cardiomyocytes, upregulated hypertrophic markers, and severe inflammation were all observed in ISO-treated mice.

It has been recently reported that the administration of SPI could reduce cardiac preload, ameliorate cardiac pump ability, and improve survival rate in patients with symptomatic heart failure and post-myocardial infarction systolic dysfunction, with obvious prognostic benefits in patients with heart failure [26]. Importantly, spironolactone can reverse isoproterenol-induced cardiomyocyte hypertrophy [27]. Here, we used SPI as a positive control for the treatment of cardiac hypertrophy. After the administration of pirfenidone, the abnormal effects on heart weight, LV wall thickness, LV volume, myocardial injury markers, cross-sectional area of cardiomyocytes, hypertrophic markers, and inflammation were significantly alleviated. This is consistent with the pharmaceutical effect of SPI, which is a promising agent for the treatment of cardiac hypertrophy [28] and is utilized as a positive control in the present study. These data confirm the therapeutic effect of pirfenidone against cardiac hypertrophy.

In recent years, the Janus kinase/signal kinase and activator of transcription (JAK/STAT) is a newly detected intracellular signal transduction pathway. It receives extracellular signal stimuli such as growth factors and cytokines to act on heterotrophic DNA fragments in the nucleus, regulate the transcription of target genes, and affect cell proliferation, differentiation, and apoptosis [29]. Relevant studies have shown the JAK2/STAT3 pathway is involved in the signal transduction process of cardiac hyperplasia and hypertrophy induced by various factors, and its activation is observed in myocardial infarction and dilated cardiomyopathy [30]. The activation of JAK2/STAT3 signaling was observed in ISO-treated mice in the present study, which was greatly reversed by pirfenidone, indicating that its protective effects might be associated with its inhibitory effect against the JAK2/STAT3 pathway. In future work, the functional mechanism of pirfenidone will be further identified by co-administering Coumermycin A1, an agonist of JAK2 [31]. Consistently, pirfenidone was reported to attenuate myocardial infarction-induced cardiac fibrosis in a rat model by activating LXR-α and controlling the feedback loop of the AT1R/p38-MAPK/RAS axis [32]. Our study provides further evidence that pirfenidone could protect cardiac function by ameliorating cardiac hypertrophy through a novel molecular mechanism.

## Conclusion

Collectively, our data reveals a therapeutic property of pirfenidone on ISO-induced cardiac hypertrophy in mice via inhibition of the JAK2/STAT3 pathway.

## Supplementary Material

Supplemental Material
