# Supplementary figures and images for: Pirfenidone attenuates cardiac hypertrophy against isoproterenol by inhibiting activation of the janus tyrosine kinase-2/signal transducer and activator of transcription 3 (JAK-2/STAT3) signaling pathway
Source: Bioengineered. 2022 May 24;13(5):12772–82. doi: 10.1080/21655979.2022.2073145 (PMC9276057; doi:10.1080/21655979.2022.2073145)

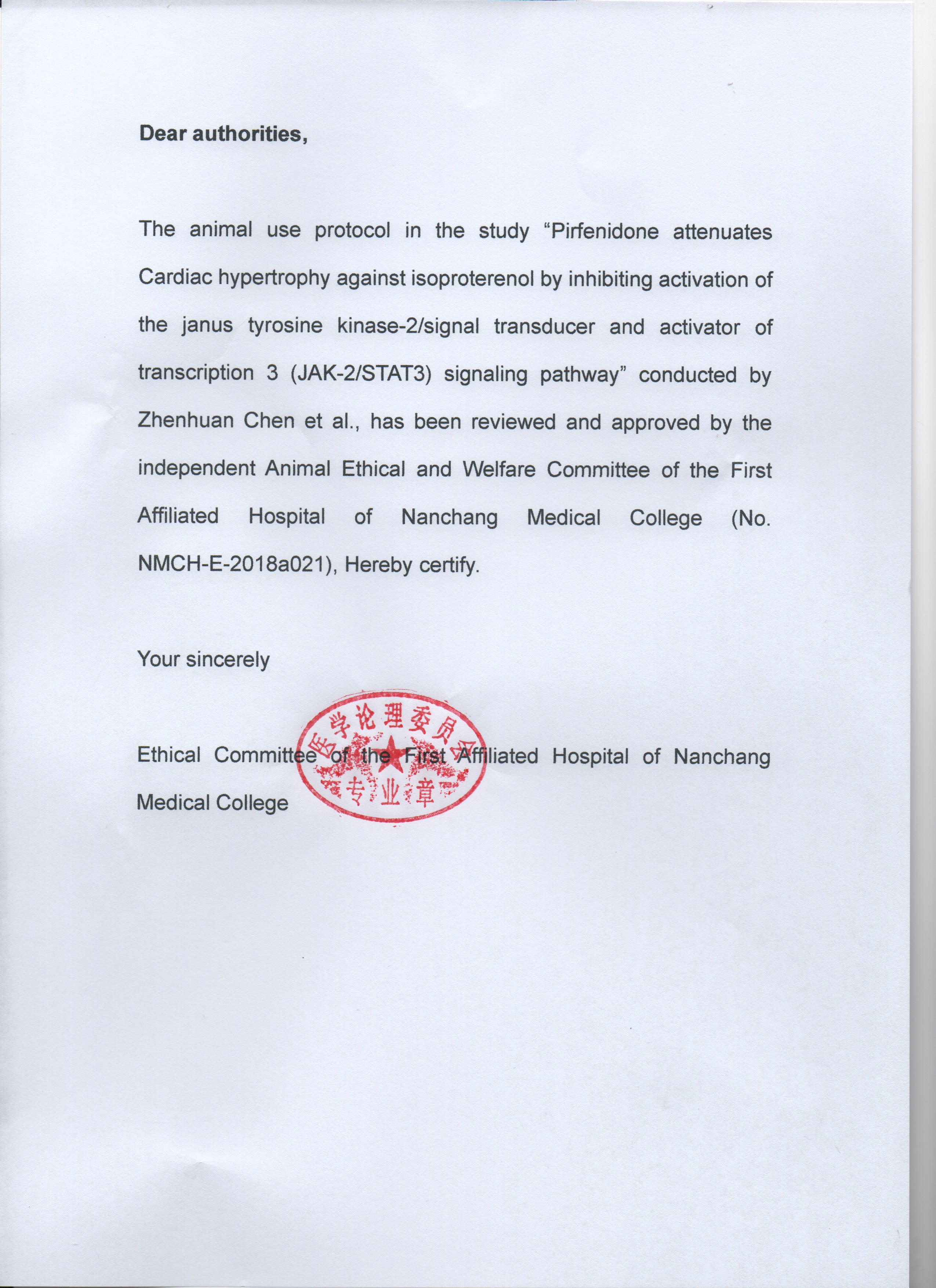

Supplement: Supplemental Material [file KBIE_A_2073145_SM5777.jpg]
